# Laser-upgraded coal tar for smart pavements in road and bridge monitoring applications

**DOI:** 10.1038/s41378-024-00670-z

**Published:** 2024-03-11

**Authors:** Jincai Huang, Man Zhang, Haoyun He, Qingang Li, Yixin Zhao, Qiulin Tan, Xining Zang

**Affiliations:** 1 Department of Mechanical Engineering, Tsinghua University, Beijing, 100084 China; 2 State Key Laboratory of Clean and Efficient Turbomachinery Power Equipment, Department of Mechanical Engineering, Tsinghua University, Beijing, 100084 China; 3 Key Laboratory for Advanced Materials Processing Technology, Ministry of Education, Beijing, 100084 China; 4 Science and Technology on Electronic Test and Measurement Laboratory, North University of China, Taiyuan, 030051 China; 5 School of Energy and Mining Engineering, China University of Mining and Technology (Beijing), Beijing, 100083 China

**Keywords:** Materials science, Electrical and electronic engineering

## Abstract

The implementation of an intelligent road network system requires many sensors for acquiring data from roads, bridges, and vehicles, thereby enabling comprehensive monitoring and regulation of road networks. Given this large number of required sensors, the sensors must be cost-effective, dependable, and environmentally friendly. Here, we show a laser upgrading strategy for coal tar, a low-value byproduct of coal distillation, to manufacture flexible strain-gauge sensors with maximum gauge factors of 15.20 and 254.17 for tension and compression respectively. Furthermore, we completely designed the supporting processes of sensor placement, data acquisition, processing, wireless communication, and information decoding to demonstrate the application of our sensors in traffic and bridge vibration monitoring. Our novel strategy of using lasers to upgrade coal tar for use as a sensor not only achieves the goal of turning waste into a resource but also provides an approach to satisfy large-scale application requirements for enabling intelligent road networks.

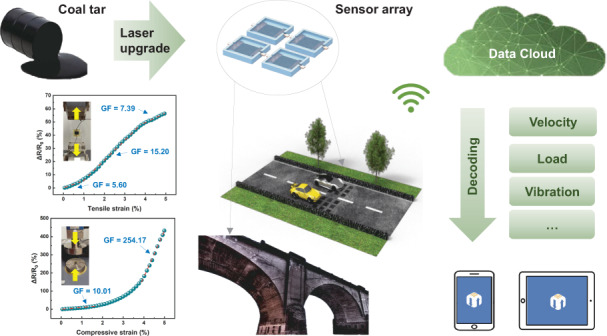

## Introduction

Strain sensors have extensive applications in weigh-in-motion systems^1,2^, structural health monitoring systems^3–5^, and pavement management systems^6,7^. These methods enable the prediction of fatigue-related damage by monitoring the horizontal strain at the bottom of the asphalt layer due to repeated loading^8,9^. Additionally, vertical strain measurements serve dual purposes by predicting ruts, a form of permanent pavement deformation caused by the passage of heavy vehicles^10^ and providing traffic monitoring data, including dynamic load and vehicle velocity assessments^11,12^.

Traditional foil strain gauges exhibit a limited working temperature range and are prone to failure when exposed to high temperatures (>180 °C) generated during asphalt mixing compaction^13^. In comparison, fiber Bragg grating sensors are better suited for pavement monitoring in challenging environments due to their exceptional corrosion resistance and resistance to electromagnetic interference^14^. However, the installation process of these devices is intricate and time-consuming^15^, because of the complex coupling of temperature and strain fields in signal processing^16,17^: they also exhibit high maintenance costs and poor integration with wireless communication technology^18^, which demand additional human effort and material resources to enable practical applications. In general, the economic and environmental costs of strain sensors should be considered for large-scale road applications, as should their performance and reliability^19^.

**Fig. 1:**
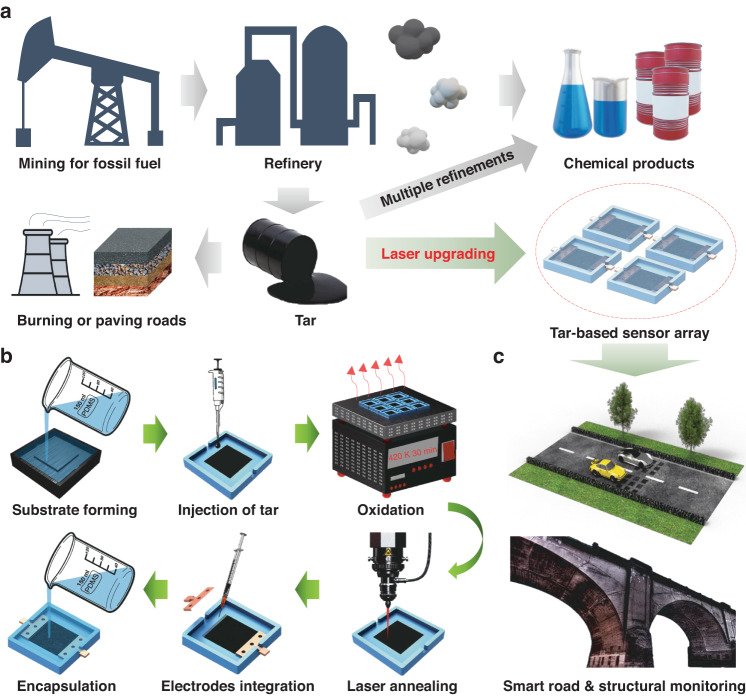
**Schematic of the traditional lifecycle and laser upgrading strategy for coal tar**. **a** The utilization methods of coal tar. **b** Laser upgrading strategy for coal tar. **c** Application scenarios of coal tar-based sensors

Coal tar, which is typically considered a low-value byproduct of coal distillation^20^, is often disposed of through burning or used in paving tar-and-chip driveways^21^ (Fig. 1a). Underutilized and undervalued coal tar contains rich chemical compounds mainly comprising aromatic hydrocarbons, making it possible to customize the tar functionality through laser annealing^22–24^. In this work, we introduce an easy-to-integrate manufacturing strategy to convert coal tar into combined sensitive and conversion elements of strain sensors packaged in polydimethylsiloxane (PDMS) for smart road and bridge vibration monitoring. With optimized laser processing parameters, the conductivity of laser-annealed coal tar (LACT) can be increased by several orders of magnitude, reaching 328.99 S/m. Then, we evaluated the horizontal and vertical strain-sensing capabilities of flexible strain sensors with specific-sized sensitive units (10 × 10 mm^2^) and employed finite-element simulation to determine the optimal placement of the sensor within the asphalt layers, such that the sensors are aligned with the strain variation zone occurring as vehicles pass by. Furthermore, we demonstrate the application of the sensors in decoding vehicle speed, wheelbase, and loading, as well as the frequency of bridge vibrations, using a system with signal collection, processing, and wireless communication functions.

Our strategy includes upgrading certain components of the road, namely the coal tar, into functional devices without introducing sensors with higher economic and environmental costs, thereby making the large-scale application of coal tar-based sensors possible. This environmentally friendly strategy explores the specific application scenarios of carbon-intensive industrial byproducts represented by coal tar, and is intended to be consistent with the global drive for carbon neutrality.

## Results and discussion

### Laser upgrading strategy for coal tar

The entire waste-to-device strategy can be summarized into six steps, including substrate formation, tar injection, oxidation, laser annealing, electrode integration, and encapsulation (Fig. 1b). PDMS was used as the substrate for the tar film, and the dimensional parameters of the substrate formed by the mold are shown in Fig. S1, with a 10 × 10 × 0.4 mm^3^ groove set in the center for the injection of tar. Coal tar primarily consists of polycyclic aromatic hydrocarbons (PAHs) with 4–6 rings^25^, and a molecular mass ranging from 217 to 300. PAHs impart viscous physical properties to coal tar, rendering it sticky and semisolid in nature^26^; as a result, the organic solvent N-methyl-pyrrolidone (NMP) was used to dilute tar to a 50% mass fraction, with a smaller contact angle for better film-forming properties than those of dichloromethane (Fig. S2). To further improve the film-forming characteristics of tar on PDMS, the PDMS substrate was subjected to surface treatment with oxygen plasma, which effectively converted the surface of the PDMS substrate from oleophobic to lipophilic. Oxidation of the tar film is a crucial step in this process. The oxidative heating causes the evaporation of small molecules and organic solvents, resulting in curing of the tar solution into a film. Moreover, oxygen-induced crosslinking of aromatic clusters obtained through low-temperature annealing contributes to structures of tar with a substantially greater degree of conjugation and stacking between aromatic sheets, increasing the conductivity after laser annealing^27^. An infrared laser was used as a local high-temperature heat source to rapidly carbonize tar thin films to achieve high conductivity (>328.99 S/m), with lower material loss (~50%) than traditional furnace annealing (>80%)^26^. The copper electrodes were then encapsulated over the substrate with PDMS, which was connected to functional laser-annealed coal tar with silver paste. The packaged LACT-based strain sensors are shown in Fig. S3.

### Optimized laser parameters for LACT

**Fig. 2:**
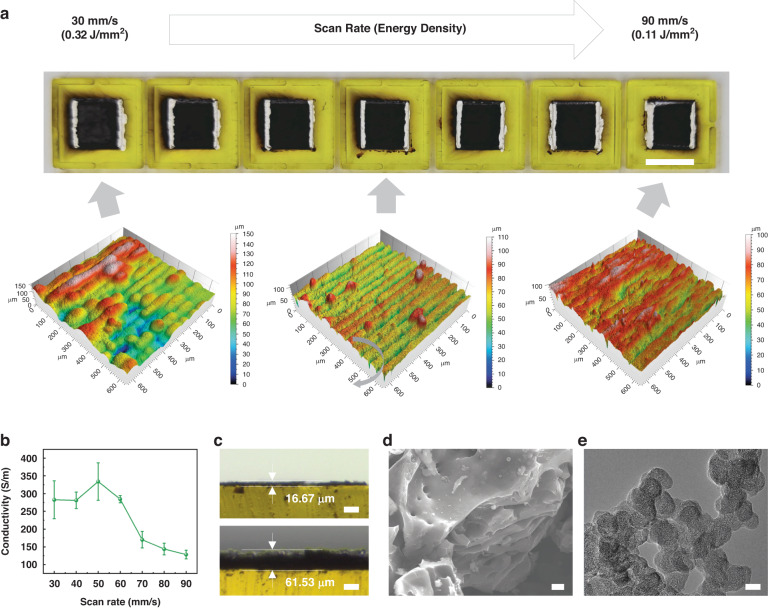
**Characterization of the morphology, conductivity, and chemical properties of the LACT.** Characterization of the morphology, conductivity, and chemical properties of the LACT. **a** Optical photos (scale bar: 10 mm), surface morphology images, and **b** conductivity of the LACT at laser scan rates ranging from 30 mm/s to 90 mm/s. **c** Optical images of the film thickness (scale bar: 50 μm), **d** SEM image (scale bar: 2 μm), and **e** TEM image (scale bar: 20 nm) of coal tar before and after laser annealing at a scan rate of 60 mm/s. The error bar represents one standard deviation

The morphology, chemistry, and conductivity of LACT are tunable by manipulating the laser processing parameters. With a laser spot size of 103.95 μm and a laser power of 1 W, we adjusted the laser scan rate from 30 to 90 mm/s to change the laser energy density^28^, defined as *P/(vd)*, from 0.32 to 0.11 J/mm^2^, where *P*, *v*, and *d* denote the laser average power, scan rate, and laser spot diameter respectively. As the scanning speed decreases, more sufficient annealing leads to an increase in conductivity. In contrast, excessive annealing occurs when the scan rate is less than 60 mm/s (Fig. 2a), which may thermally deform the substrate. The surface morphology damage caused by excessive annealing limits further improvement in the conductivity of the LACT film (Fig. 2b). The thickness of the initial oxidized coal tar film is approximately 16.67 microns and then increases to 61.53 microns after optimized laser annealing (Fig. 2c). The layered structure of LACT was observed by scanning electron microscopy (Fig. 2d) and compared to that of the coal tar film before annealing (Fig. S4), and the transmission electron microscopy image illustrates the para-crystalline nanoscale (<20 nm) graphitic sheets of LACT, which are similar to carbon black and amorphous hydrocarbons (Fig. 2e).

Raman spectroscopy is commonly used to characterize the structure of carbon materials by analyzing the extracted feature peaks^29^. Typically, the intensity ratio of the D peak (near 1350 cm^–1^) to the G peak (near 1600 cm^–1^) is used to evaluate the defects, disordering, and *sp*^2^/*sp*^3^ content. In addition, the intensity of the 2D peak (near 2750 cm^–1^) reflects the graphitic stacking of the materials^30–32^. The similar Raman spectra shown in Fig. S5 for the coal tar films annealed at different scan rates indicate that the structural change in the LACT is not significant for the applied energy density range. The conductivity is mainly affected by the integrity of the surface conductive networks. As depicted in Fig. S6, the formation of a grid-like surface morphology can be attributed to the laser scanning line distance. Considering the morphology, conductivity, and processing efficiency, a scan rate of 60 mm/s was adopted for the sensors.

### Performance testing for LACT-based strain sensors

**Fig. 3:**
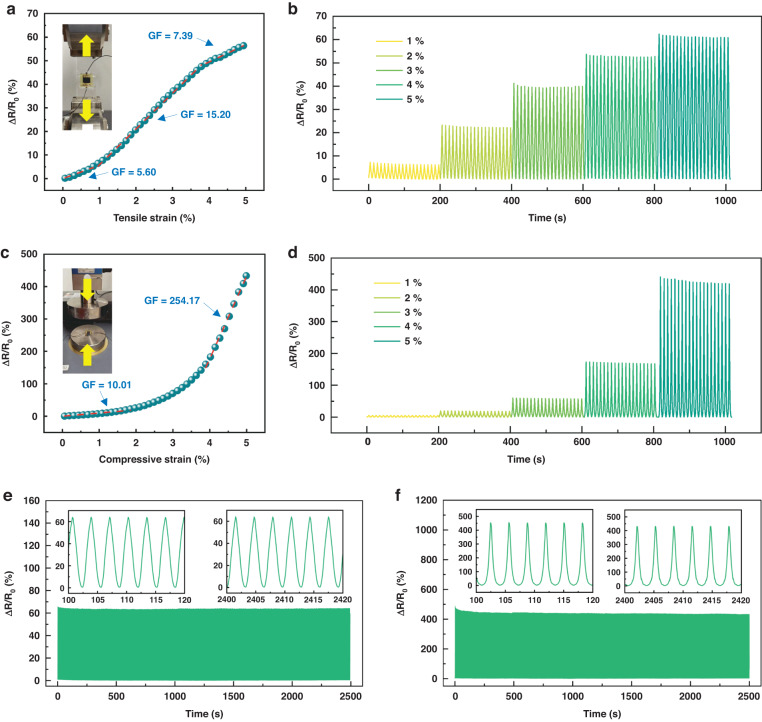
**Performance of LACT-based sensors under mechanical stimulus.** **a** Relative resistance change in response to tensile strain in the length direction under quasistatic loading rate and **b** ~10 s/cycle. **c** Relative resistance change in response to compressive strain in thickness direction under quasistatic loading rate and **d** ~10 s/cycle. Cyclic tests of the sensors at 5% strain for more than 800 cycles toward **e** tensile strain and **f** compressive strain (~3.1 s/cycle). Insets: Waveforms from the 100th to 120th second and from the 2400th to 2420th second

To demonstrate the output from the mechanical stimulus, we recorded relative changes in resistance as a function of applied strain in both the length and thickness (Fig. 3a, c), and the corresponding stress-strain relationships are shown in Fig. S7. To assess the sensitivity of the assembled device as a strain-gauge sensor, the gauge factor (GF), defined as GF *= (∆R/R*_*0*_*)/ε*, where *∆R/R*_*0*_ and *ε* denote the relative resistance change and applied strain respectively, was measured with a gradual increase in strain up to 5%. If the applied strain is greater than 5%, there may be a permanent deviation between the sensor resistance and the initial resistance after unloading, which can be attributed to local damage to conductive networks (Fig. S8). The piecewise linearity of the resistance response to strain can be observed in Fig. 3a, c: 0% ~ 1% with GF = 5.60, 1~4% with GF = 15.20, 4~5% with GF = 7.39 for tension and 0~2% with GF = 10.01, 4~5% with GF = 254.17 for compression. Compression in the thickness direction has a much greater sensitivity than stretching in the length direction. Figure 3b, d shows the resulting data, which represent the variations in resistance under cyclic strain loading, reaching peak strain values of 1%, 2%, 3%, 4%, and 5%. The stability of the sensor can be proven by over 800 strain loading cycles with a maximum strain of 5% (Fig. 3e, f). The resistance response waveforms remain almost unchanged over 800 strain cycles, indicating favorable stability and repeatability in both the compression and tension directions. Optical images of the LACT-based sensors under different strains are shown in Fig. S9. To demonstrate the sensor’s tolerance to humidity, we tested the relative resistance changes of the sensor as a function of applied strain under different humidity conditions (Fig. S10). The sensor performance is not sensitive to humidity. Although the packaging material (PDMS) may exhibit different mechanical properties at different humidity levels, the electrical signal output of the sensitive layer does not significantly change under the same strain loading.

### Applications for traffic and bridge vibration monitoring

**Fig. 4:**
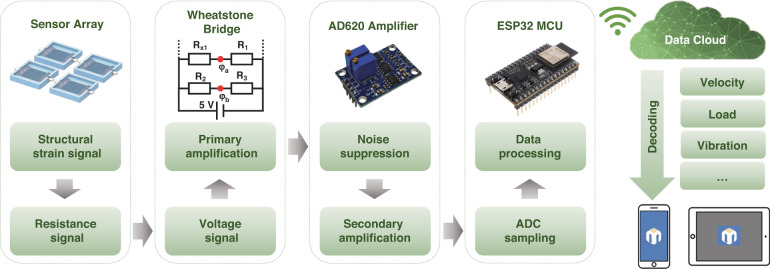
Block diagram of signal acquisition, processing, and wireless communication for LACT-based sensors

The large-scale application of sensors on roads and bridges requires sensors to be economical, practical, and environmentally friendly and to call for efficient signal acquisition and transmission methods. Figure 4 shows the signal acquisition and transmission process based on the proposed Internet of Things technology, which is suitable for application scenarios with many sensor terminals. This process comprises three steps: signal acquisition, data transmission, and data decoding. The first two steps are completed at the sensor terminals, including analog-to-digital conversion, signal amplification, noise suppression, and wireless communication, while the third step is executed remotely to obtain road, bridge, and vehicle information, promoting the development of the structural health monitoring system. Additional implementation details are provided in the “Materials and method” section.

Furthermore, we utilized our LACT-based sensors to measure the speed of a moving car model and the corresponding vibration of a bridge model to demonstrate the sensor’s application potential for monitoring roads and bridges. The decoding of vehicle speed and load leverages the vertical strain-sensing capacity of the sensor, while the decoding of bridge vibration frequency is achieved through its horizontal strain-sensing capability.

**Fig. 5:**
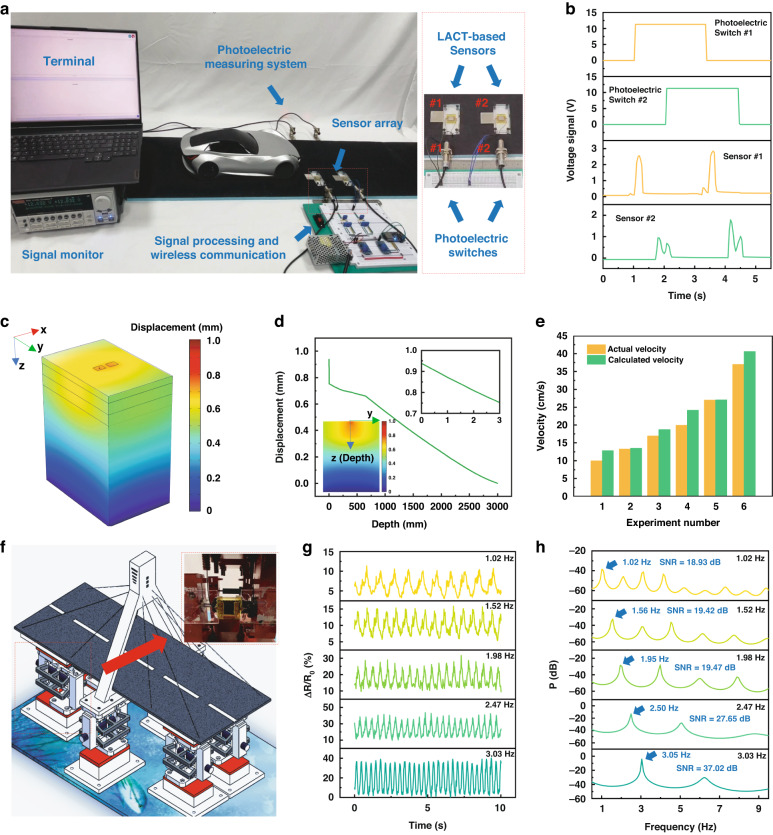
**The application of LACT-based sensors for traffic and bridge vibration monitoring.** **a** Experimental system for verifying vehicle monitoring. **b** The signals collected by the photoelectric speed measurement system and the LACT-based sensors at 10 cm/s. **c**, **d** Finite-element simulation of semirigid base asphalt pavement model to determine the placement of the sensors. **e** Comparison of the speed measurements between LACT-based sensors and photoelectric switches. **f** The miniaturized bridge model for vibration monitoring. Inset: Attachment position of the sensor. **g** Time-domain signals collected by sensors at different vibration frequencies. **h** The power spectra of the signals for the extraction of the vibration frequency

A speed measurement system based on sensor arrays is shown in Fig. 5a; this system consists of two LACT-based sensors; two photoelectric switches for obtaining the actual velocity; a system integrated with signal acquisition, processing, and wireless communication functions; and a terminal. The LACT-based sensors can capture the signals occurring when each tire passes by, where the pulse signal peak, the time delay of adjacent pulse signals, and the time delay of the front and rear sensors’ pulse signals correspond to the load, wheelbase, and speed of the vehicle respectively. These signals were collected and wirelessly uploaded to the cloud computing environment using the process shown in Fig. 4 to decode the vehicle speed, and the pulse signals sampled are shown in Fig. 5b. The recorded signals are often not ideal pulse signals, but rather contain several pulse peaks. This phenomenon is reasonable because the signal waveforms reflect the dynamic process of contact between the car and the sensors when passing by, which includes the vibration of the vehicle, the impact caused by vehicle contact and departure sensors, and other dynamic information about the vehicle. Through peak finding and supporting calculations, we calculate the vehicle speed, load, and wheelbase from the collected sensing signals (Figs. 5e and S11). The original signal waveforms used to construct the speed calibration chart (Fig. 5e) are shown in Fig. S12. Moreover, we conducted tests on the sensors in practical environments. To withstand the heavy load generated by people and vehicles, we used acrylic gaskets to adjust the strain range applied by the sensor and avoid damage (Fig. S15). The two sensors were placed 80 cm apart. With the stiffness regulators installed, the sensors have both high stability and sensitivity when vehicles pass by (Video S4). It is more reasonable to package LACT-based sensors in Marshall specimens, a technique that also facilitates embedding the sensor directly into the road as part of it. Improved techniques for embedding LACT-based sensors into roads and achieving predetermined functions will be the focus of our next work.

Due to the use of experimental models, it is necessary to demonstrate the feasibility of using these sensors in practical application scenarios. As a result, we developed a finite-element simulation to determine the optimal placement of the sensor within the asphalt layers, aligning it with the strain variation zone as vehicles pass by. The finite-element model uses a semirigid pavement structure^33^ that is based on the following two assumptions: the structural layers of the road are continuous, homogeneous, and isotropic linear elastic materials; and the structural layers of the road are completely continuous in the vertical direction. The corresponding simulation results are shown in Fig. 5c, d, with LACT-based sensors installed one centimeter below the road surface. The parameters used in the simulation are shown in Table S1, and additional implementation details are provided in the Materials and method section. In addition, we tested the temperature characteristics of the sensors, as shown in Fig. S13.

Furthermore, we attached sensors to the shock absorber of a miniaturized bridge model that was designed for earthquake simulation to verify the bridge vibration monitoring capabilities (Fig. 5f). Figure 5g shows the sensor signals collected at different frequencies, which are subsequently processed in the cloud to decode the vibration information via Fourier transform. We used the Burg algorithm-based autoregressive model to estimate the power spectrum of the collected time-domain signals^34^, with optimized orders according to the minimum final prediction error (FPE) and Akaike information criterion (AIC) (Fig. S14). The power spectra of the sensing signals at different frequencies are shown in Fig. 5h. The results indicate that the vibration information can be accurately decoded and that the signal-to-noise ratio (SNR) still exceeds 18 dB even at low frequency (1.02 Hz). Videos of the vehicle and bridge vibration monitoring experiments can be found in the supplementary materials.

## Conclusion

We explored a laser upgrading strategy for coal tar as a strain sensor, and the conductivity of the laser-annealed coal tar reached 328.99 S/m. The LACT-based sensors exhibit a strain capacity of more than 5% under both tension in the length direction and compression in the thickness direction, while the sensitivity varies greatly: the maximum GF is 15.20 with a strain of 1~4% for tension, and the maximum GF is 254.17 with a strain of 4~5% for compression. Furthermore, we designed a supporting system including data acquisition, processing, and wireless communication functions to enable the application of the sensors in traffic and bridge vibration monitoring. By decoding the pulse signals of the sensing array, the loading, wheelbase, and speed of the vehicle are accurately estimated. Moreover, power spectrum estimation of sensor signals can quickly determine the frequency of bridge vibrations with a SNR greater than 18 dB. This environmentally friendly and cost-effective sensor, which is fabricated by easily scaled manufacturing methods, offers great potential for large-scale application in smart roads and bridges.

## Materials and method

### Preparation of LACT-based sensors

The PDMS base and the curing agent (DC184, Dow Corning) were mixed in a 10:1 ratio, and vacuum defoaming was performed for 10 min. The PDMS substrates were formed based on customized aluminum alloy molds by heating for 15 min at 150 °C. Silicon-based release agents can be used to assist in the efficient release of PDMS substrates. Coal tar (Anshan Iron and Steel Group Co., Ltd) was diluted to a 50% mass fraction using NMP as a solvent to reduce viscosity, after which 30 μL of coal tar solution was transferred to a PDMS substrate cleaned by oxygen plasma. A coal tar film with a thickness of approximately 16 μm can be obtained after low-temperature annealing in air (150 °C for 30 min). An infrared fiber pulse laser (1064 nm, pulse width <15 ps, Berlin Laser) was used to anneal the coal tar film with an average power of 1 W, a laser spot diameter of 103.95 μm and a line spacing of 50 μm. After laser annealing, copper electrodes were connected to the laser-annealed coal tar using conductive silver paste, which was subsequently packaged with PDMS. To facilitate testing, wires were soldered onto the copper electrodes after encapsulation.

### Finite-element simulation of the semirigid pavement model under static loads

To investigate the strain endured by the sensors in real-world scenarios to showcase the viability of sensor integration into roads, we made certain assumptions under static load conditions. Assuming an even distribution of the vehicle’s weight on its wheels and a rectangular contact surface between the wheels and the road, a standard axle load of 100 kN for a dual-wheel group was considered. The contact area for each wheel and the road surface was calculated as 213 × 167 mm² (*P* = 0.7 MPa). Utilizing the elastic layering theory, a model was developed in COMSOL Multiphysics, incorporating a surface layer of 15 cm, a subbase layer of 25 cm, a subseal layer of 25 cm, and a subgrade layer of 235 cm. These layers were then integrated based on assumptions of material continuity and small deformation^35^. The specific structural parameters and material properties of each layer are detailed in Table S1. Fixed constraints were applied to the bottom surface of the subgrade layer, and a uniformly distributed 0.7 MPa load was then applied to the double rectangular contact area of the road. The strain-depth relationship of the sensor embedded in the road surface at the center of the rectangular contact area was analyzed under static load conditions, revealing that the sensor experiences a maximum strain of around 6% under extreme loads, aligning well with the operational range of the strain sensor.

### System with data acquisition, processing, and wireless communication functions

Wheatstone bridges were used to convert the resistance-changing signals of the LACT-based sensors into voltage signals with primary amplification. Then, the signal outputs from the bridge were subjected to noise suppression and secondary amplification by the AD620 module, to meet the input requirements of the micro control unit’s analog-to-digital converter. We use ESP32 as the microcontroller and implement wireless communication based on the Wi-Fi module. The collected signals are simply processed in ESP32 and then uploaded to the data cloud. The decoding of signals was completed on the data cloud to obtain the vehicle’s speed, loading, bridge vibration, and so on. This signal acquisition, processing, and wireless transmission process can be easily extended to large-scale road sensor applications.

### Traffic and bridge vibration monitoring experiments

The experiments are all based on signal acquisition, processing, and wireless communication systems. Use photoelectric switches to detect the speed of the vehicle as the standard speed while using two LACT-based sensors arranged in front and rear to obtain information on vehicle passage. The speed at which a vehicle passes can be calculated based on the sensor spacing (10 cm) and the difference in response time between the two sensors. In addition, the wheelbase of the vehicle can be calculated based on the time difference between the passing signals of the front and rear wheels, and the loading is reflected by the peak value of the signals.

LACT-based sensors were attached to the vibration absorber to obtain vibration information about the bridge. Vibration initiators are used to simulate the vibration of bridges subjected to longitudinal seismic waves. The sensor can obtain time-domain signals of vibration (relative resistance changes) at 1.0, 1.5, 2.0, 2.5, and 3.0 Hz. We performed power spectrum estimation on the collected signals to obtain the frequency domain characteristics of the signals. A Burg algorithm-based autoregressive model was adopted, and it is necessary to choose a suitable order of the model for reliable spectral estimation. A low order can make it difficult to distinguish spectral peaks, while a high order may result in false peaks. The order of the models was optimized according to the minimum FPE and AIC.

1 $${\rm{FPE}}\left(k\right)={\rho }_{k}\frac{N+\left(k+1\right)}{N-\left(k+1\right)}$$

2 $${\rm{AIC}}\left(k\right)=N\mathrm{ln}\left({\rho }_{k}\right)+2k$$

where *N* represents the length of the sampled time-domain signals, and ${\rho }_{k}$ denotes the minimum prediction error power of k-order model for signals. The orders chosen were 40, 28, 20, 15, and 12 for signals of 1.0, 1.5, 2.0, 2.5, and 3.0 Hz, respectively, to obtain relatively small values for the two criteria. The order optimization of autoregressive models and the power spectrum estimation with the autoregressive model based on the Burg algorithm are implemented using Statistics and Machine Learning Toolbox, MATLAB.

### Supplementary information

Supplementary Information

Wireless communication in speed measurement

Sensor layout for speed measurement

Bridge vibration monitoring

Sensor testing in real environments

## Data Availability

All the data needed to evaluate the conclusions in the paper are presented in the paper and/or the Supplementary Information.
