# Supplementary material for: Laser-upgraded coal tar for smart pavements in road and bridge monitoring applications
Source: Microsyst Nanoeng. 2024 Mar 11;10:34. doi: 10.1038/s41378-024-00670-z (PMC10928128; doi:10.1038/s41378-024-00670-z)
Supplement: Supplementary file 1 — Supplementary Information [file 41378_2024_670_MOESM1_ESM.docx]

**Supplementary Information**

**Laser-upgraded coal tar for smart pavements towards road and bridge monitoring applications**

Jincai Huang^1, 2, 3^, Man Zhang^1, 2, 3^, Haoyun He^4^, Qingang Li^1, 2, 3^, Yixin Zhao^5^, Qiulin Tan^4^, Xining Zang^1, 2, 3^^*^

^1^ Department of Mechanical Engineering, Tsinghua University, Beijing, 100084, China

^2^ State Key Laboratory of Clean and Efficient Turbomachinery Power Equipment, Department of Mechanical Engineering, Tsinghua University, Beijing, 100084, China

^3^ Key Laboratory for Advanced Materials Processing Technology, Ministry of Education, Beijing, 100084, China

^4^ Science and Technology on Electronic Test and Measurement Laboratory, North University of China, Taiyuan, 030051, China

^5^ School of Energy and Mining Engineering, China University of Mining and Technology (Beijing), Beijing, 100083, China

^*^ Corresponding author, email: [xzang@tsinghua.edu.cn](mailto:xzang@tsinghua.edu.cn)


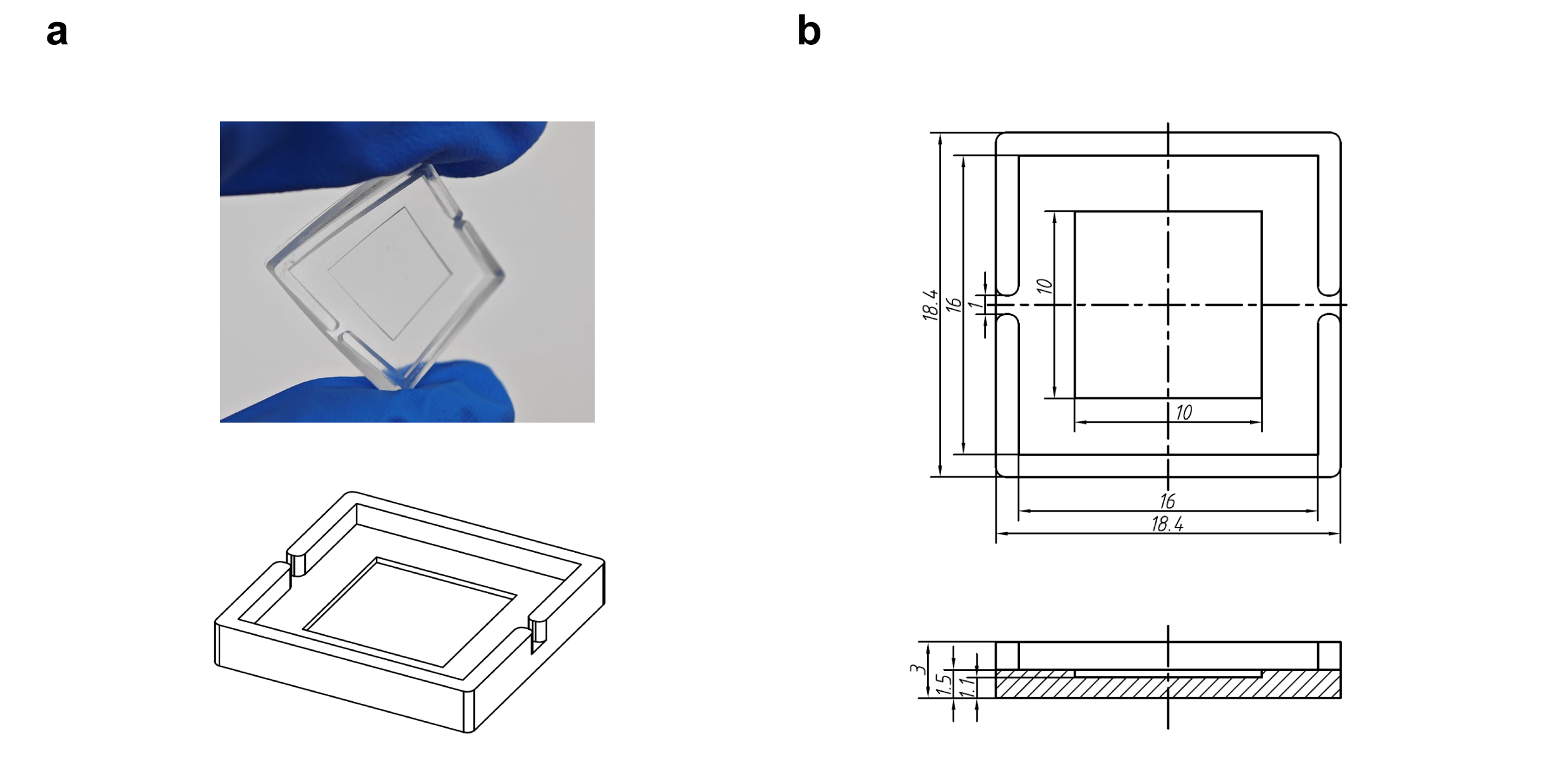


**Fig. S1: a** Optical image and isometric drawing of the polydimethylsiloxane (PDMS) substrate. **b** Specific dimensions of the substrate.


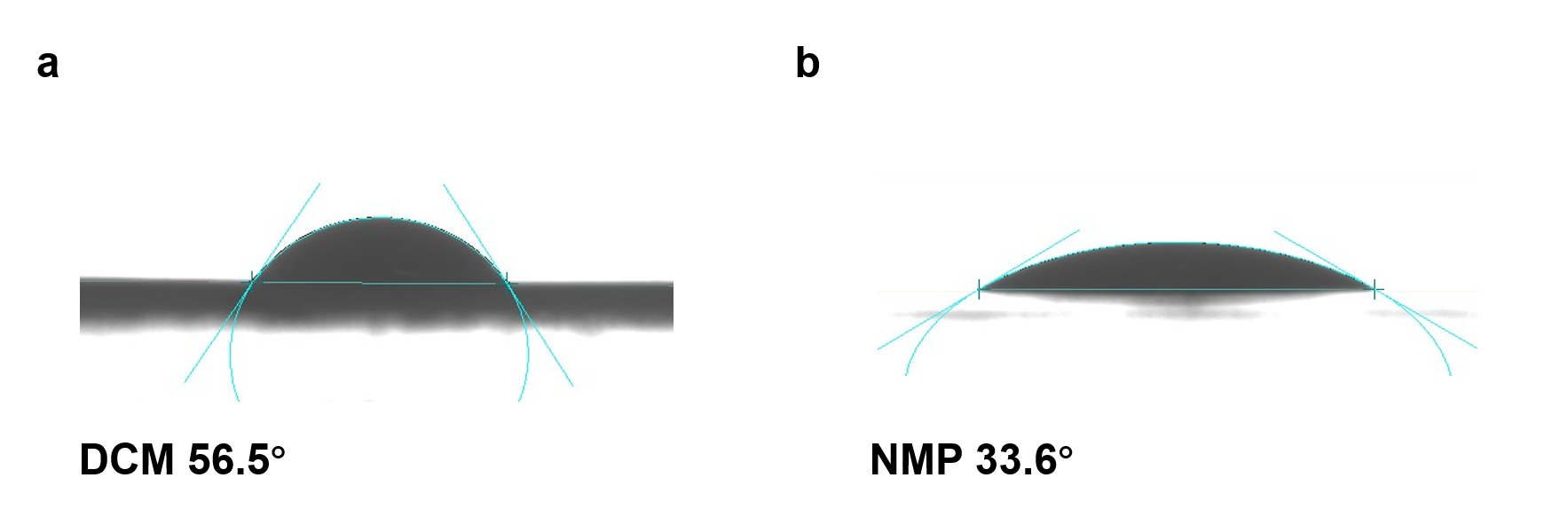


**Fig. S2: Contact angle between coal tar solution and PDMS. a** Coal tar dichloromethane solution. **b** Coal tar N-methyl-pyrrolidone solution. Both are 50% mass fraction.


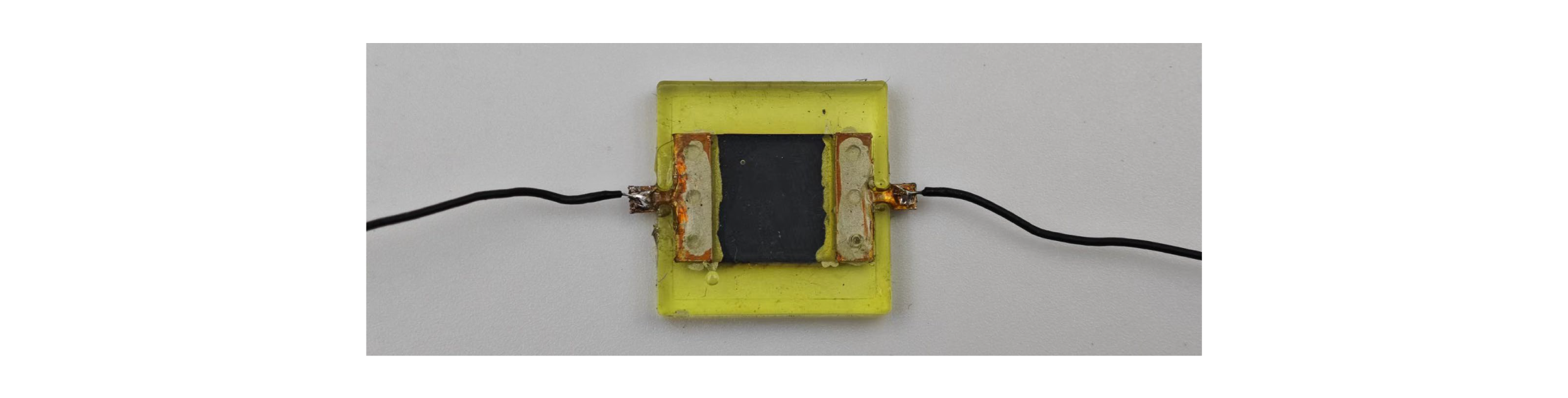


**Fig. S3: Optical image of the encapsulated LACT-based sensor.**

**
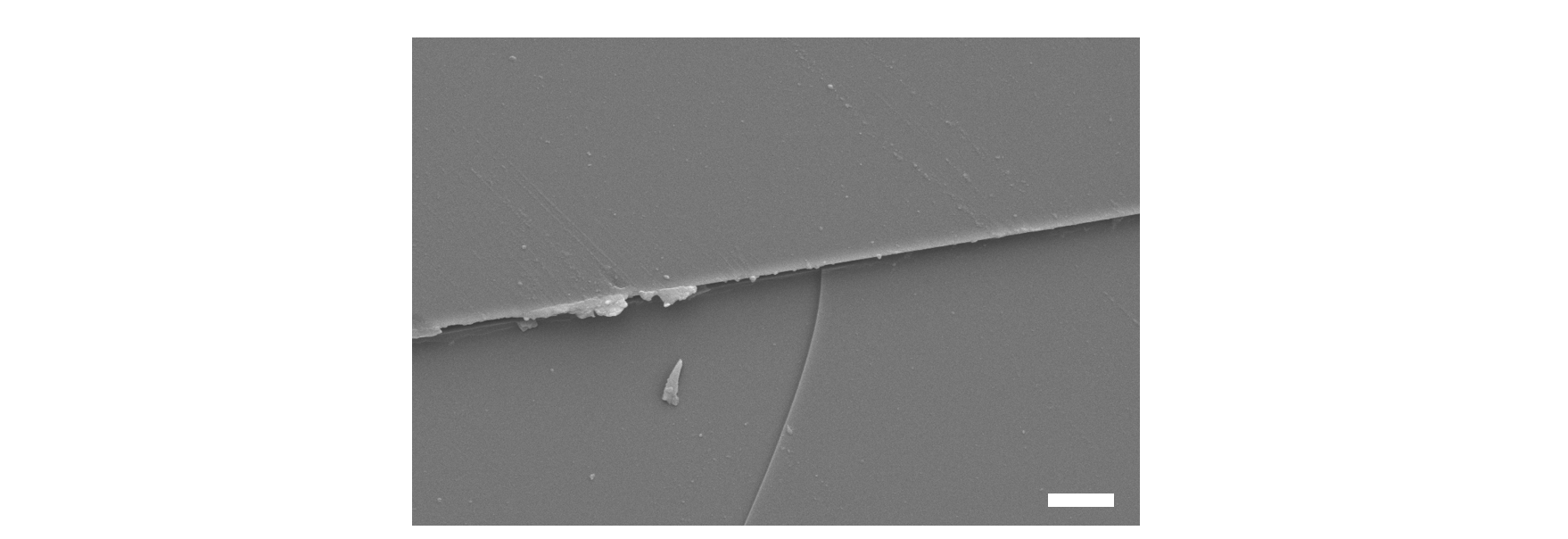
**

**Fig. S4:** **Scanning electron micrographs of oxidized coal tar film before laser annealing.** Scale bar: 2 μm.


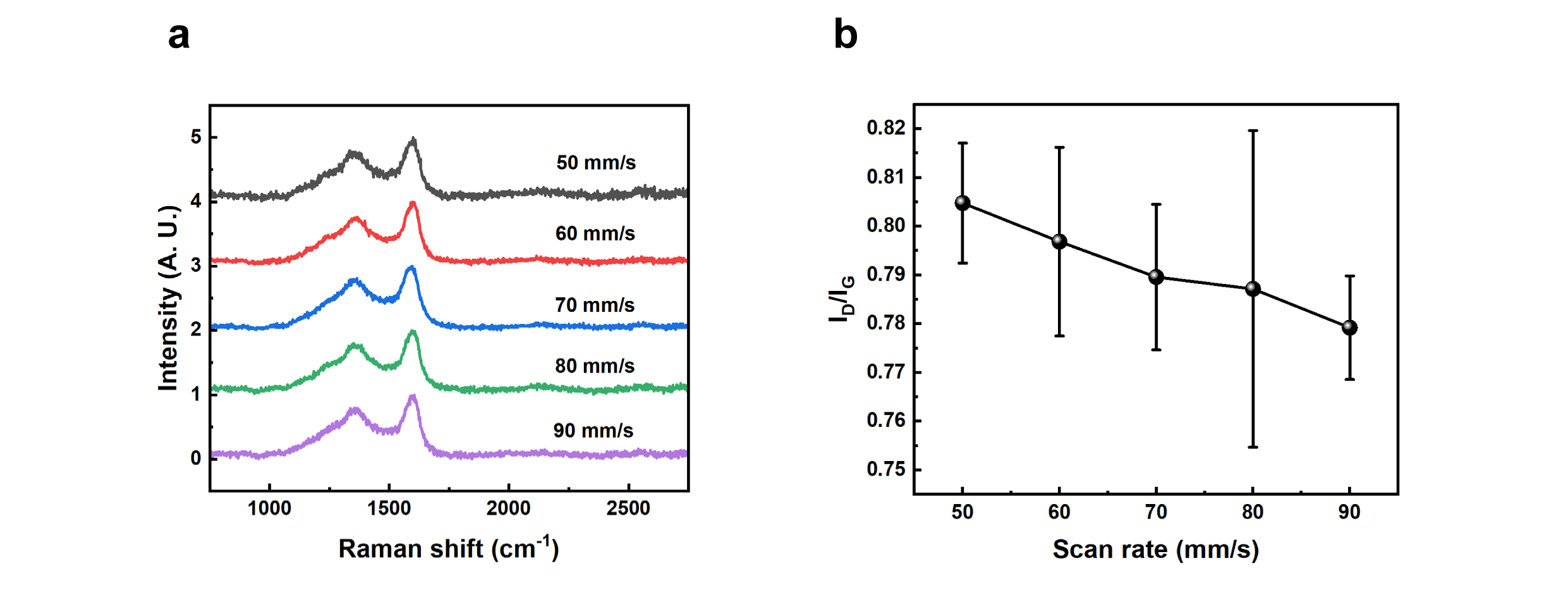


**Fig. S5: a** Raman spectra and **b** the ratio of D peak intensity to G peak intensity of the laser-annealed coal tar at different scan rates.


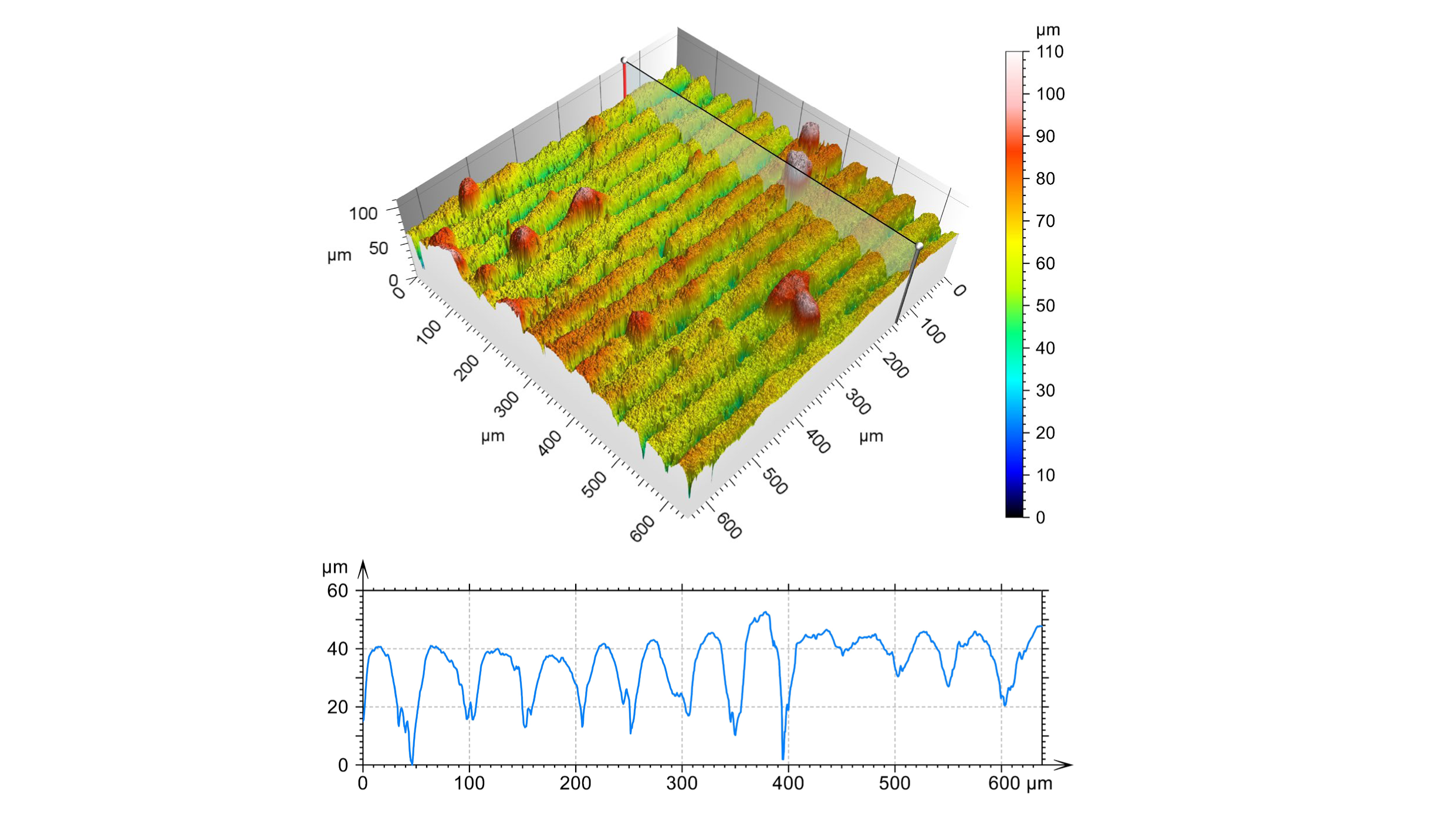


**Fig. S6: Surface morphology map of the laser-annealed coal tar at the scan rate of 60 mm/s.**


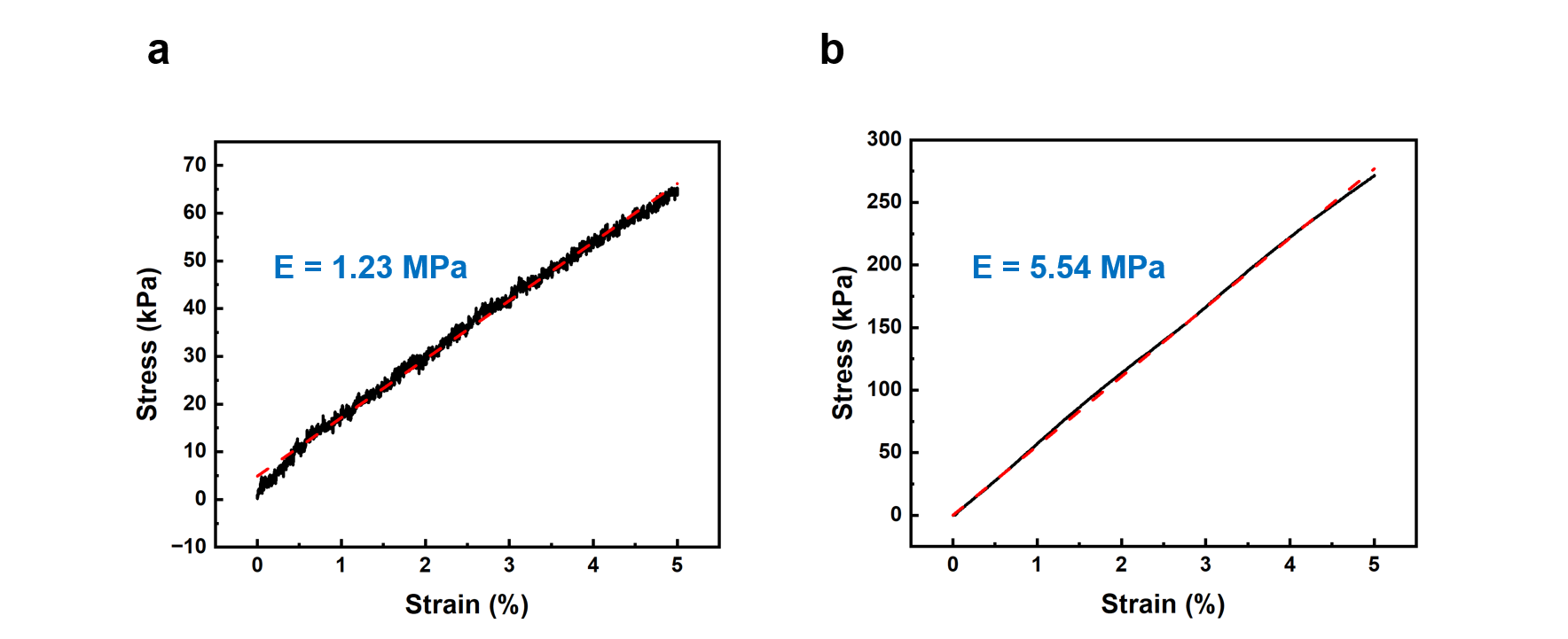


**Fig. S7:** Stress-strain curves under **a** tension and **b** compression experiments. The loading rate is 0.01 mm/s, the mass ratio of pre-polymer (base) and cross-linker (curing agent) is 10:1, and the temperature of the experimental environment is 298 K.

**
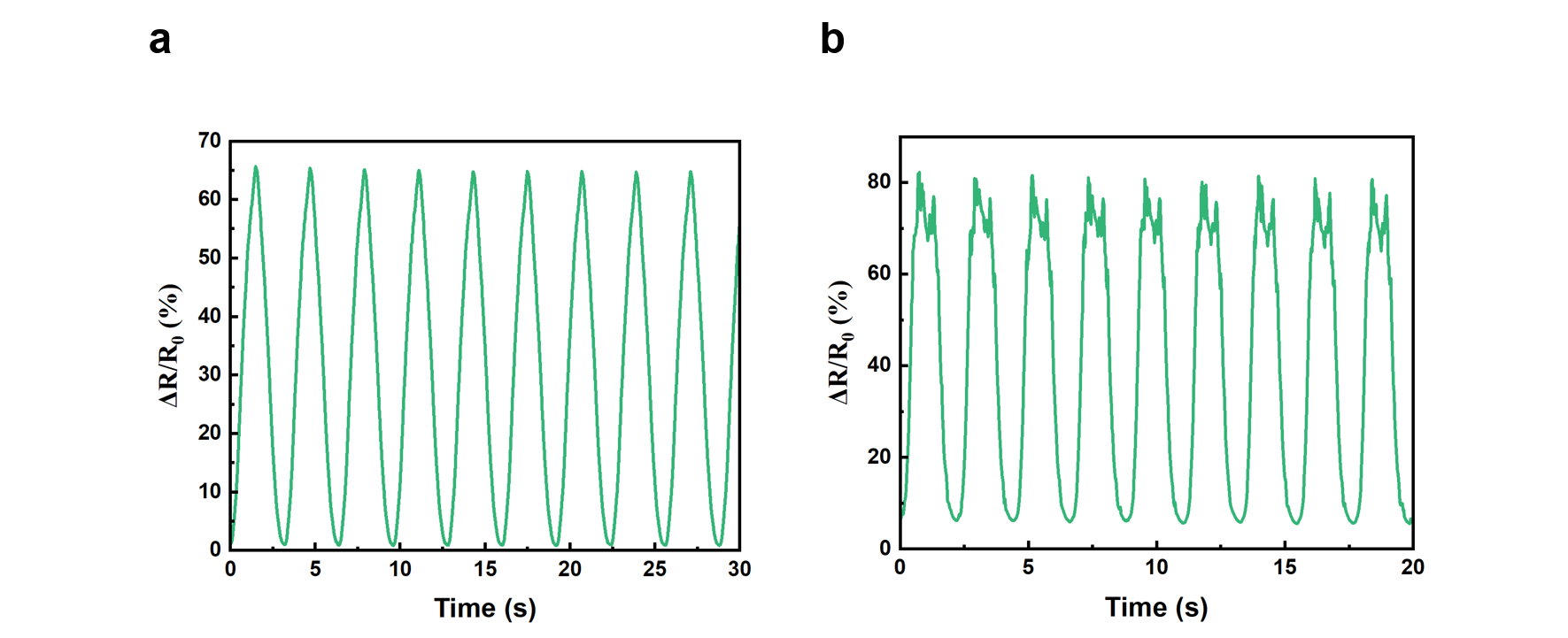
**

**Fig. S8: Waveforms of relative resistance changes intercepted in strain tensile cycling experiments at 5% and 6% strain. a** Maximum strain of 5%, ~ 3.1 s/cycle. **f** Maximum strain of 6%, ~ 2 s/cycle.

From the cyclic tests of the sensor, we found that when the maximum cyclic strain is greater than 5%, the relative resistance change signals of the sensor may become unstable, and there are many ‘burrs’ in the signal waveform. This phenomenon may be caused by excessive local microcracks. In addition, the valley value of the waveform in Fig. S8b has increased, which means that it may be more difficult for the sensor to recover to the initial ‘0’ value after unloading, which corresponds to the initial resistance value. This means that the sensor has undergone permanent structural changes and needs to be recalibrated. To avoid this situation in the experiment, we chose a maximum strain of 5%, which is a conservative choice.

**
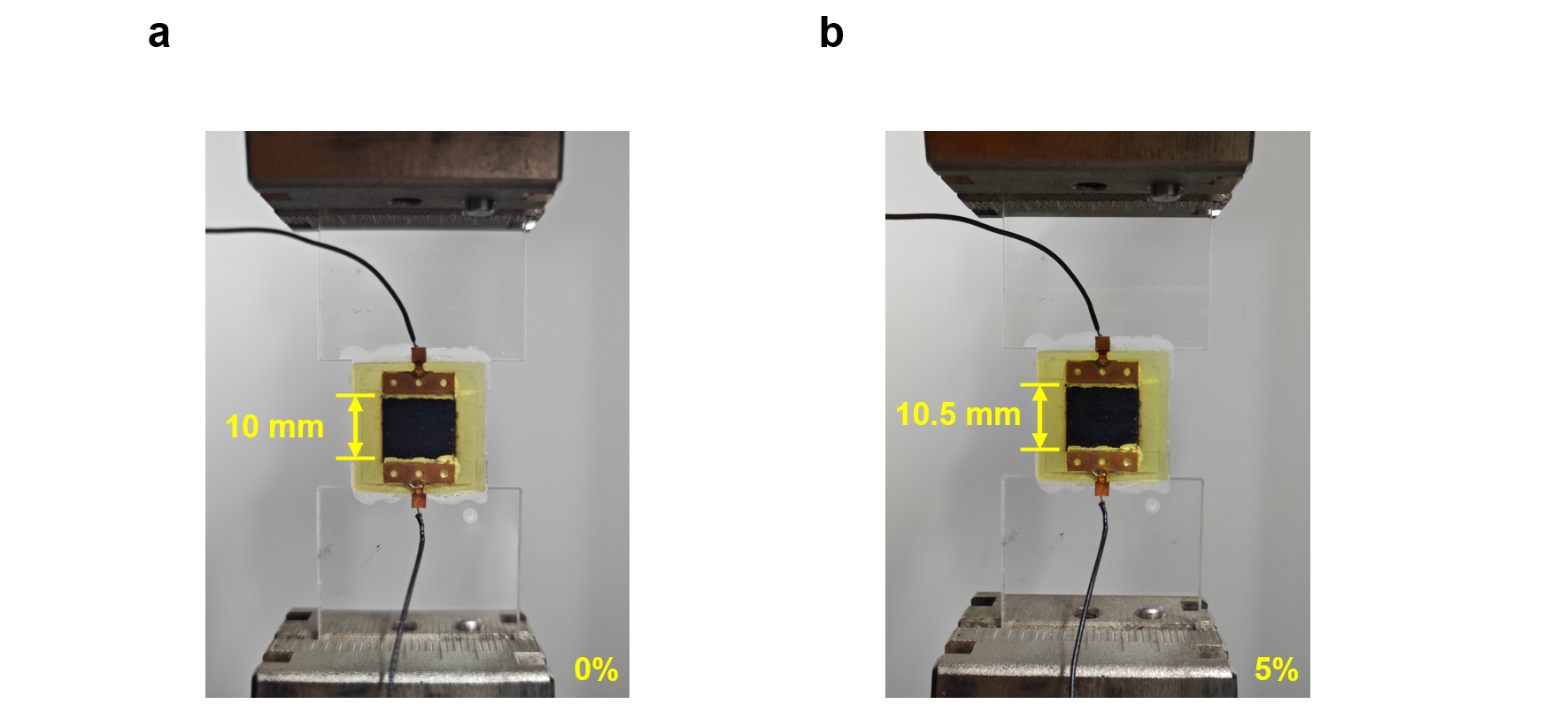
**

**Fig. S9:** Optical images of the sensor in tensile experiments at **a** 0% strain and **b** 5% strain.

To perform a tensile test on the sensor, it is necessary to stick the sensor onto two substrates for easy clamping of the stretching mold. The initial spacing of the substrates is 10 mm, corresponding to the edge length of the sensor’s sensitive layer. When using a mechanical stretching machine (Shimadzu AGX-V) to load the substrate spacing to 10.5 mm, the strain of the sensor is considered to be 5%.

**
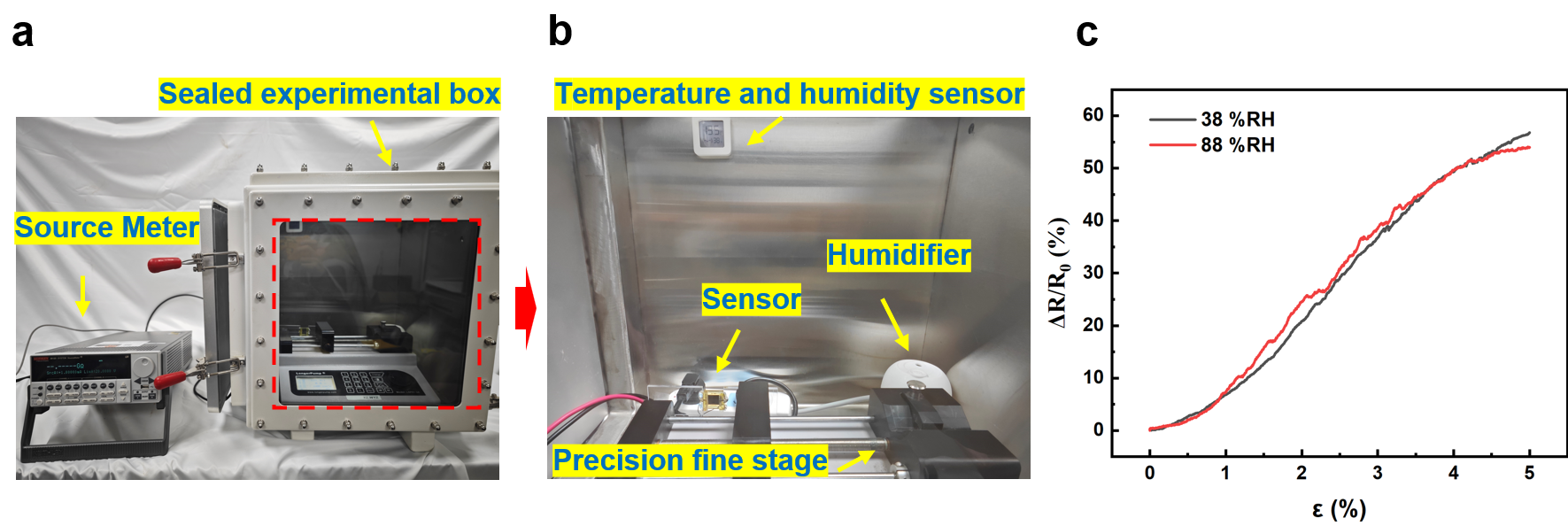
**

**Fig. S10: Demonstration of the sensor’s tolerance to humidity.**


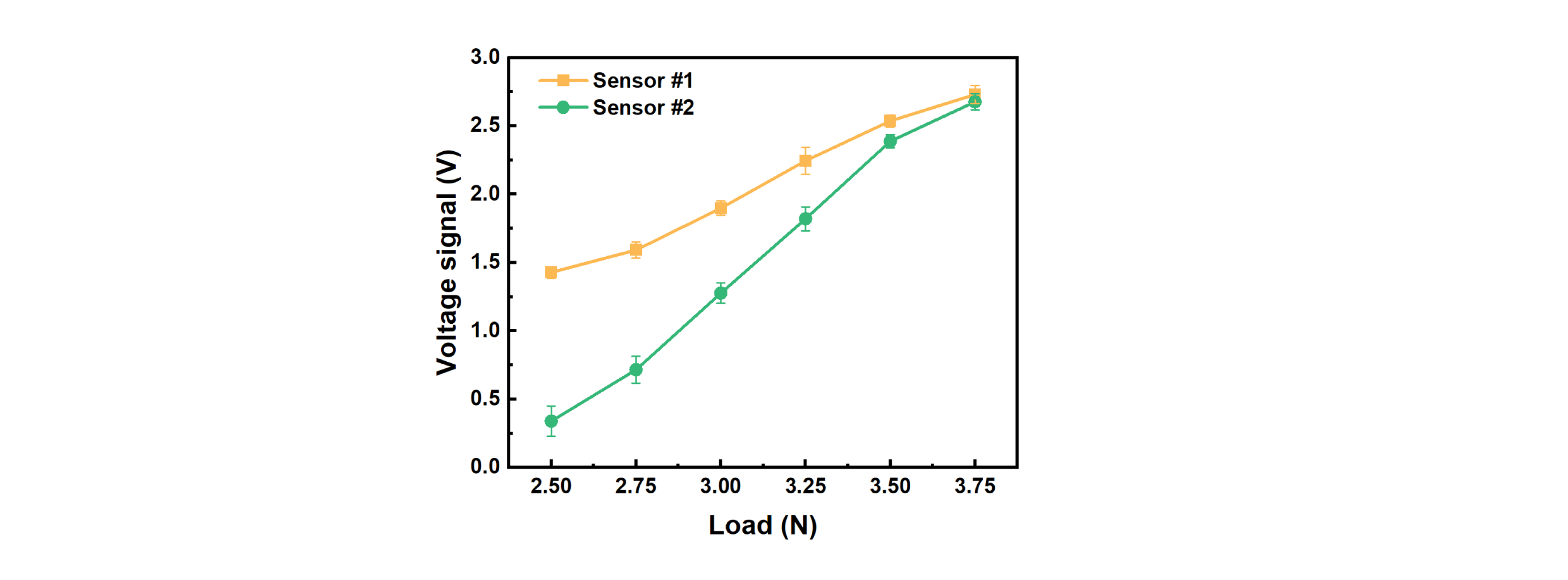


**Fig. S11: The response of sensor output voltage signals to load.**


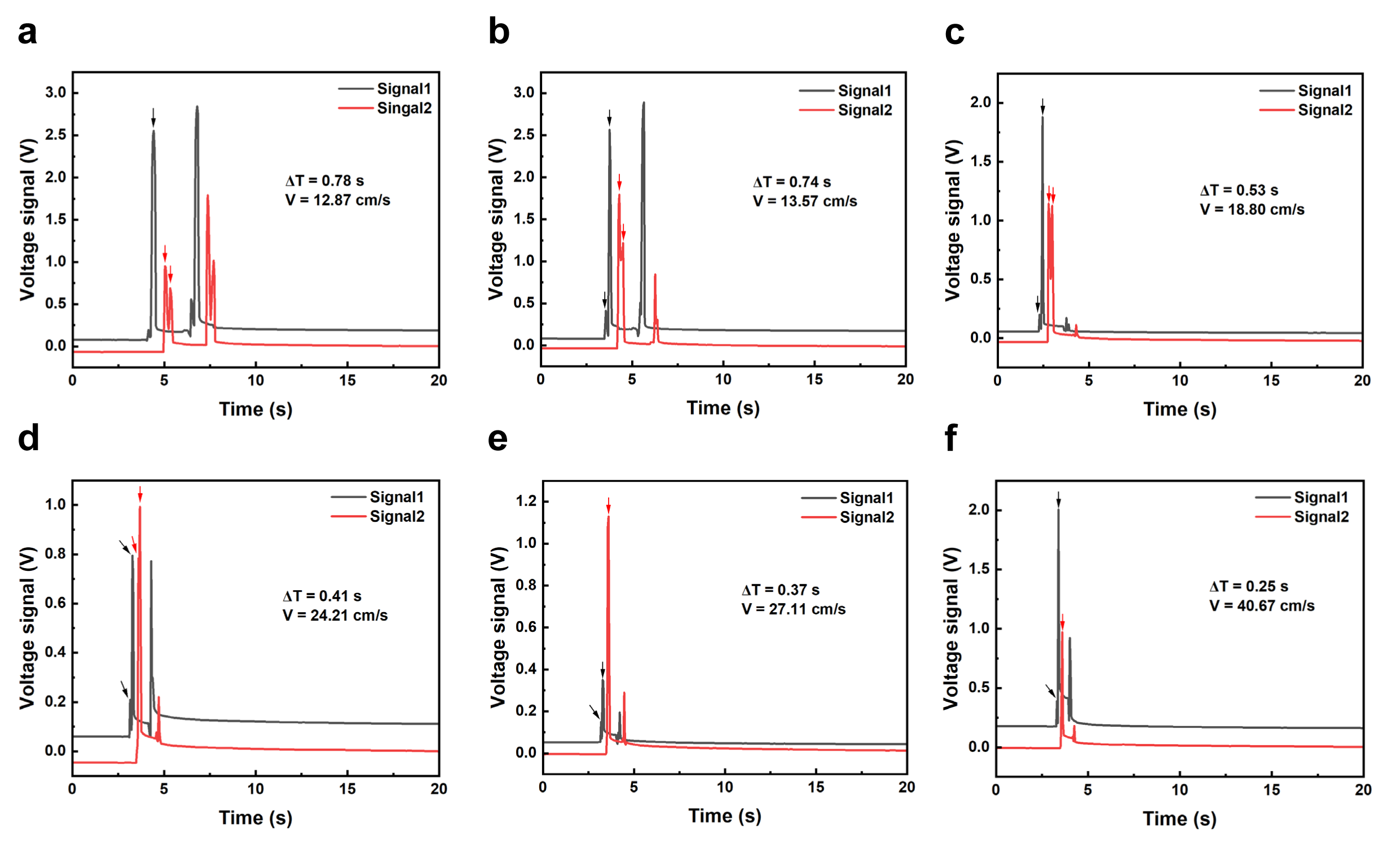


**Fig. S12:** **Waveform diagrams of sensor signals.** Output signals of LACT-based sensors placed at intervals of 10cm as the vehicle model passed at **a** 10.0, **b** 13.3, **c** 16.9, **d** 20.0, **e** 27.0, **f** 37.0 cm/s.

**Table S1: Parameters used in finite element models.**

| **Component** | **Length**  **(mm)** | **Width**  **(mm)** | **Thickness**  **(mm)** | **Poisson’s**  **ratio** | **Elasticity modulus**  **(MPa)** | **Density**  **(kg/m^3^)** |
| --- | --- | --- | --- | --- | --- | --- |
| Surface layer | 1600 | 2400 | 150 | 0.3 | 1800 | 2400 |
| Sub-base layer | 1600 | 2400 | 250 | 0.25 | 1200 | 2100 |
| Sub-seal layer | 1600 | 2400 | 250 | 0.25 | 400 | 2000 |
| Subgrade layer | 1600 | 2400 | 2350 | 0.35 | 50 | 1800 |
| Contact area | 213 | 167 |  |  |  |  |

The tire pressure on the ground is 0.7 MPa.


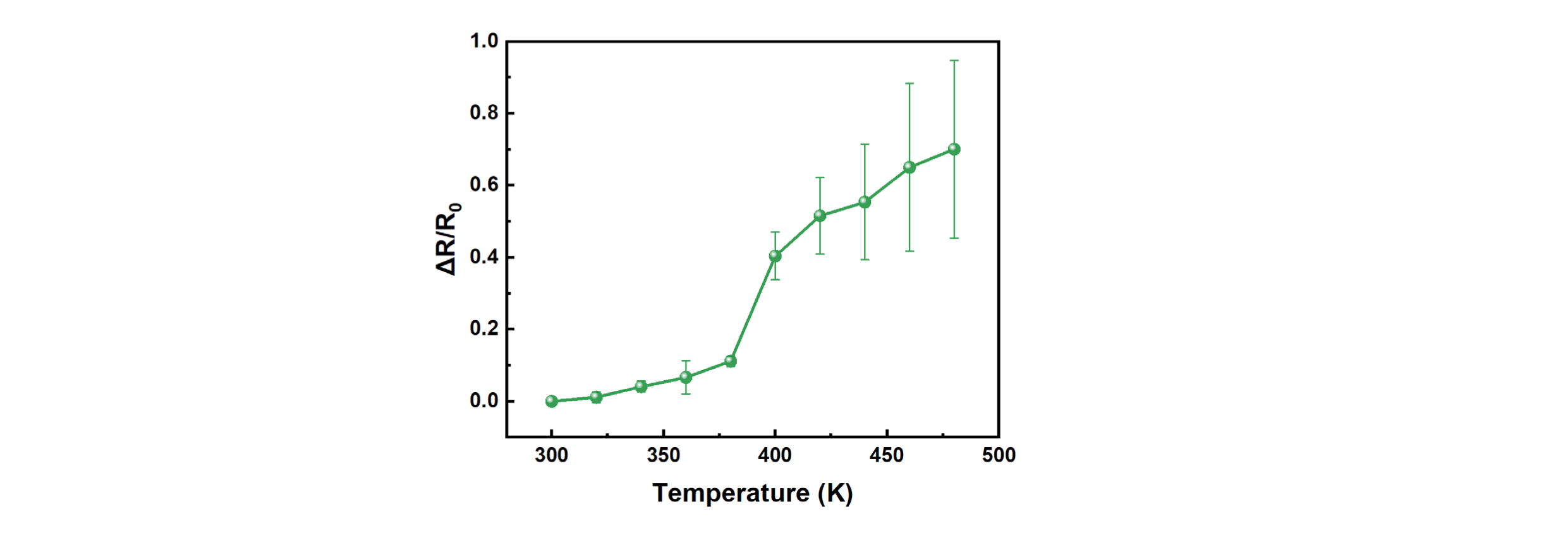


**Fig. S13:** **The relative resistance change responding to temperature.** The error bar represents one standard deviation.


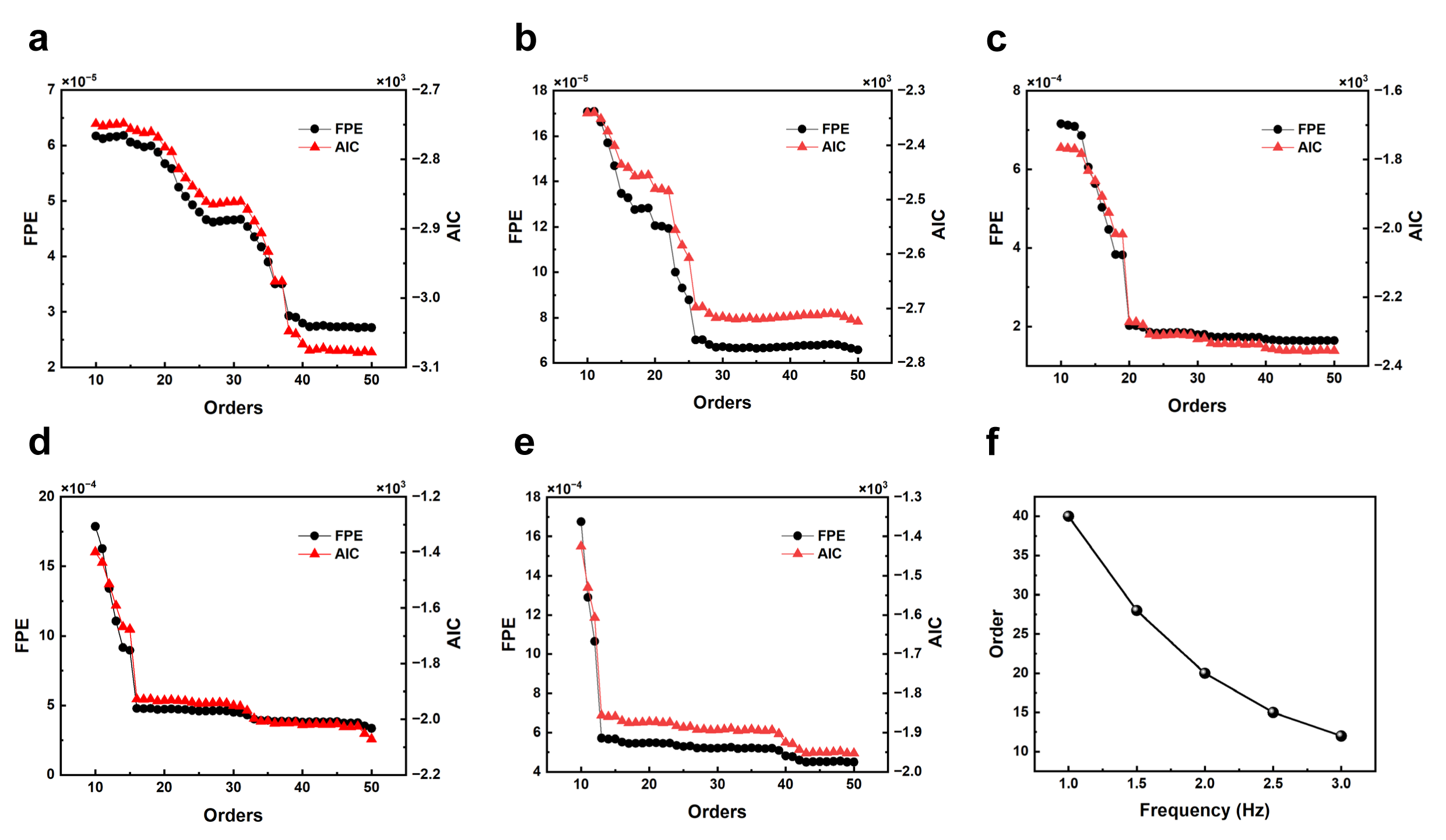


**Fig. S14: The order optimization of the autoregressive models according to minimum final prediction error (FPE) and Akaike information criterion (AIC). a-e** correspond to input signals of 1.0 Hz, 1.5 Hz, 2.0 Hz, 2.5 Hz, and 3 Hz respectively. **f** The order chosen with the frequency of the input signal**.**

**
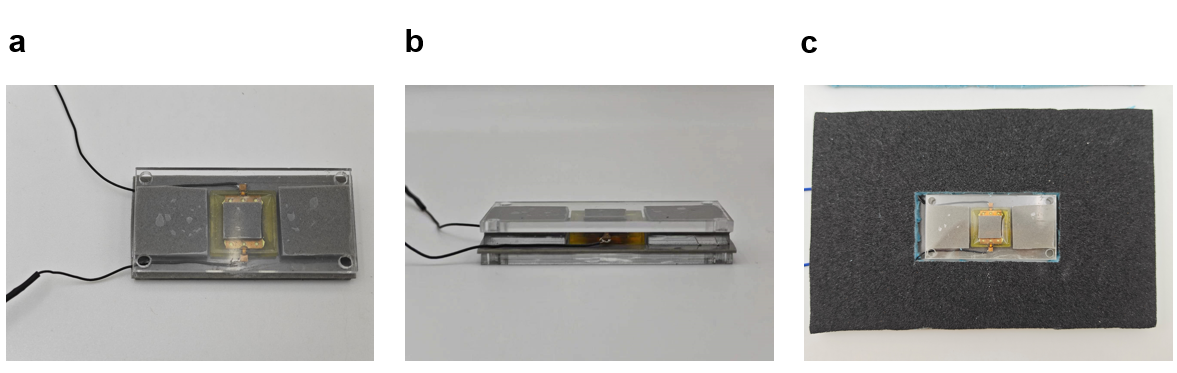
**

**Fig. S15: Sensors used for testing in real environments.**
